# Theoretical insights of codoping to modulate electronic structure of $\hbox {TiO}_2$ and $\hbox {SrTiO}_3$ for enhanced photocatalytic efficiency

**DOI:** 10.1038/s41598-020-72195-0

**Published:** 2020-09-21

**Authors:** Manish Kumar, Pooja Basera, Shikha Saini, Saswata Bhattacharya

**Affiliations:** Department of Physics, Indian Institute of Technology Delhi, New Delhi, 110016 India

**Keywords:** Electronic properties and materials, Photocatalysis, Photocatalysis, Computational chemistry, Electronic structure

## Abstract

$\hbox {TiO}_2$ and $\hbox {SrTiO}_3$ are well known materials in the field of photocatalysis due to their exceptional electronic structure, high chemical stability, non-toxicity and low cost. However, owing to the wide band gap, these can be utilized only in the UV region. Thus, it’s necessary to expand their optical response in visible region by reducing their band gap through doping with metals, nonmetals or the combination of different elements, while retaining intact the photocatalytic efficiency. We report here, the codoping of a metal and a nonmetal in anatase $\hbox {TiO}_2$ and $\hbox {SrTiO}_3$ for efficient photocatalytic water splitting using hybrid density functional theory and ab initio atomistic thermodynamics. The latter ensures to capture the environmental effect to understand thermodynamic stability of the charged defects at a realistic condition. We have observed that the charged defects are stable in addition to neutral defects in anatase $\hbox {TiO}_2$ and the codopants act as donor as well as acceptor depending on the nature of doping (p-type or n-type). However, the most stable codopants in $\hbox {SrTiO}_3$ mostly act as donor. Our results reveal that despite the response in visible light region, the codoping in $\hbox {TiO}_2$ and $\hbox {SrTiO}_3$ cannot always enhance the photocatalytic activity due to either the formation of recombination centers or the large shift in the conduction band minimum or valence band maximum. Amongst various metal-nonmetal combinations, $\hbox {Mn}_\text {Ti}\hbox {S}_\text {O}$ (i.e. Mn is substituted at Ti site and S is substituted at O site), $\hbox {S}_\text {O}$ in anatase $\hbox {TiO}_2$ and $\hbox {Mn}_\text {Ti}\hbox {S}_\text {O}$, $\hbox {Mn}_\text {Sr}\hbox {N}_\text {O}$ in $\hbox {SrTiO}_3$ are the most potent candidates to enhance the photocatalytic efficiency of anatase $\hbox {TiO}_2$ and $\hbox {SrTiO}_3$ under visible light irradiation.

## Introduction

Semiconductor-based photocatalysts are seeking the attention ascribed to their potential in utilizing the solar energy to cater to the current energy demand of the world and also, serve the purpose of pollutant degradation^1–9^. Anatase $\hbox {TiO}_2$ and $\hbox {SrTiO}_3$ are two of the metal oxides that can be used in photocatalytic water splitting^4,10–24^ owing to their suitable band edge positions. However, they could only exploit the UV irradiation of the solar spectrum attributed to their wide band gap of $\sim \,3.2$ eV. This leads to the low photocatalytic efficiency and limits their application at a commercial level. An efficient photocatalyst should have congenial band gap such that it is wide enough to straddle the redox potential of a desired compound and narrow enough to absorb the visible light of the solar spectrum. However, despite after significant research endeavors, finding the same has never been easy both experimentally as well as theoretically. Therefore, there is justified interest to reduce the band gap and induce visible light response by means of doping with metals^25–31^, nonmetals^32–40^ or their combination^41–47^. Although the doping can tune the band gap^10–18^, many a time the band edge positions also get changed and the localized deep trap states occur in forbidden region leading to faster recombination. As a consequence, the photocatalytic efficiency gets degraded. Therefore, we need to ensure the suitable band edge positions as well as the passivation of midgap trap states (i.e. recombination centers).

The conduction band minimum (CBm) lies $\sim$ 0.4 eV and 0.8 eV above the reduction potential of water for anatase $\hbox {TiO}_2$^48^ and $\hbox {SrTiO}_3$^49^, respectively, concomitant with the reduction of water to produce hydrogen under UV irradiation. The transition metal dopant shifts the CBm downwards [towards valence band maximum (VBM)] and hence results in deterioration of reduction power. On the other hand, in case of nonmetal dopant, the band gap reduction takes place by elevating the VBM. However, many a time localized deep trap states appear in forbidden region in both the aforementioned cases. Moreover, the nonmetal doped systems are unstable against exposure to light irradiation^50^. Therefore, despite the visible light absorption, none of the monodopants (be it metal or nonmetal) are usually suitable for photocatalytic water splitting. This has motivated us to codope the system. The codoping of a metal and a nonmetal is one of the prominent solutions to passivate the trap states and form the shallow impurity states^50^. In addition, the codoping enhances the solubility of nonmetal doping in anatase $\hbox {TiO}_2$ or $\hbox {SrTiO}_3$^42^. Note that for maximum efficiency, the band gap should be $\sim$ 2 eV^51,52^. With the aid of codoping, the band gap could be tailored such that it induces visible light absorption while retaining the redox powers and thus, enhances the photocatalytic efficiency^53^. Note that the experimental synthesis of individual monodopants (i.e. N, S, Rh and Mn) in $\hbox {TiO}_2$ as well as $\hbox {SrTiO}_3$ have already been done^38,39,54–61^. A very few experimental studies also exist on N-Mn and S-Mn codoped $\hbox {TiO}_2$ as well^62,63^. However, for the codoping (viz. N-Mn, N-Rh, S-Mn and S-Rh) in bulk $\hbox {SrTiO}_3$ and (N-Rh, S-Rh) in anatase $\hbox {TiO}_2$, any experimental or theoretical reports are hitherto unknown.

In this article, we have studied the role of codopants (N-Mn, N-Rh, S-Mn and S-Rh) in anatase $\hbox {TiO}_2$ and $\hbox {SrTiO}_3$ for enhancing the photocatalytic efficiency. We have shown in Ref.^64,65^ that the substitutional doping is more favorable than the interstitial in $\hbox {TiO}_2$ as well as $\hbox {SrTiO}_3$. Therefore, we have chosen only the substitutional positions (i.e. metal at Ti or Sr site and nonmetal at O site) for codoping. First, we have determined the thermodynamic stability of codoped $\hbox {TiO}_2$ and $\hbox {SrTiO}_3$ under the framework of hybrid density functional theory (DFT)^66,67^ and ab initio atomistic thermodynamics at realistic conditions [temperature (*T*), partial pressure of oxygen $(p_{\text {O}_2})$ and doping]^68^. Next, the electronic structure has been analyzed to get insight about the defect states. Further, we have calculated the optical properties to compare the spectral response of all the defected configurations w.r.t. pristine counterpart. To explore the best set of codoped combinations for photocatalytic water splitting, the band edge alignment has been carefully carried out. Finally, under codoping, the effective mass has been determined to understand the effect on mobility of photogenerated charge carriers.

## Results

### Stability of codoped $\hbox {TiO}_2$ and $\hbox {SrTiO}_3$

The stability has been determined by calculating the defect formation energy using hybrid DFT and ab initio atomistic thermodynamics^68–70^. The defect configuration, having minimum formation energy, is the most stable defect. For a defect X with charge state *q*, the formation energy $\text {E}_\text {f}(\text {X}^{q})$ is evaluated as follow^64,68,71^:

1 $$\begin{aligned} \text {E}_\text {f}(\text {X}^{q})&= \text {E}_\text {tot}(\text {X}^{q}) - \text {E}_\text {tot}(\text {pristine}^{0}) - \sum _{i} {n}_i\mu _{i}\\&\quad + {q}(\mu _\text {e} + \text {VBM} + \Delta \text {V}), \end{aligned}$$

where $\text {E}_\text {tot}(\text {X}^{q})$ and $\text {E}_\text {tot}(\text {pristine}^{0})$ are the energies of defected supercell (charged) and pristine supercell (neutral), respectively, calculated using hybrid DFT. ${n}_i$ is the number of species *i* added to or removed from the pristine supercell and $\mu _{i}$’s are the corresponding chemical potentials, which is selected with reference to the total energy ($\text {E}_\text {tot}({i}^{0})$) of species *i*. Therefore, $\mu _{i} = \Delta \mu _{i} + \text {E}_\text {tot}({i})$ (*i* = N, S, O, Mn, Rh, Sr, or Ti), where $\Delta \mu _{i}$’s are chosen according to the environmental growth conditions. $\mu _\text {e}$ is the chemical potential of the electron, which is the energy required to exchange electrons between the system and the electrons’ reservoir. It is varied from VBM to CBm of the pristine supercell. $\Delta \text {V}$ is the core level alignment between pristine neutral and defected supercell.

**Figure 1:**
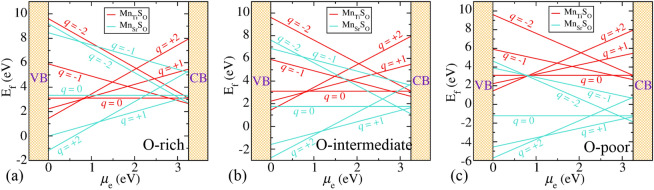
Formation energy of Mn-S related defect in $\hbox {SrTiO}_3$ at (**a**) O-rich ($\Delta \mu _\text {O}=0$ eV), (**b**) O-intermediate ($\Delta \mu _\text {O}=-1.58$ eV), and (**c**) O-poor ($\Delta \mu _\text {O}=-4.55$ eV) conditions.

In our previous findings, we have seen that the substitutional defect is more favorable in comparison to interstitial and also observed that the monodopants in general are not suitable for photocatalytic application^64,65^. Therefore, we have considered here the codoped cases [metal (Rh or Mn) substituted at Ti or Sr site, and non-metal (N or S) substituted at O site].

To know the stability of different charged states at different environmental growth conditions, first we have shown the 2D formation energy plot for Mn-S related defect in $\hbox {SrTiO}_3$ (see Fig. 1a–c). For, ${\text {Mn}_\text {Sr}}{\text {S}_\text {O}^{q}}$ defect in $\hbox {SrTiO}_3$, the formation energy is given by:

2 $$\begin{aligned} \text {E}_\text {f}({\text {Mn}_\text {Sr}}{\text {S}_\text {O}^{q}})&=\text {E}_\text {tot}({\text {Mn}_\text {Sr}}{\text {S}_\text {O}^{q}}) - {\text {E}_\text {tot}}({\text {SrTiO}_3^{0}}) + \mu _\text {O} - {\mu _\text {S}}\\&\quad + {\mu _\text {Sr}} - \mu _\text {Mn} + {q}(\mu _\text {e} + \text {VBM} + \Delta \text {V}) \end{aligned}$$

Here, $\mu _\text {O} = \Delta \mu _\text {O} + \frac{1}{2}\left( \text {E}_\text {tot}(\text {O}_2) + \frac{\text {h}\nu _\text {OO}}{2}\right)$, where the last term is the zero point energy of $\hbox {O}_2$ molecule. And for, ${\text {Mn}_\text {Ti}}{\text {S}_\text {O}^{q}}$ defect in $\hbox {SrTiO}_3$, the formation energy is given by:

3 $$\begin{aligned} {\text {E}_\text {f}}({\text {Mn}_\text {Ti}}{\text {S}_\text {O}^{q}})&={\text {E}_\text {tot}}({\text {Mn}_\text {Ti}}{\text {S}_\text {O}^{q})} - {\text {E}_\text {tot}}({\text {SrTiO}_3^{0}}) + {\mu _\text {O}} - {\mu _\text {S}}\\&\quad + {\mu _\text {Ti}} - {\mu _\text {Mn}} + {q}({\mu _\text {e}} + \text {VBM} + \Delta \text {V}) \end{aligned}$$

The chemical potential of a species incorporates the effect of temperature and pressure^72^. For oxygen, the $\Delta {\mu _\text {O}}$ as a function of temperature (*T*) and the partial pressure of oxygen ($p_{\text {O}_2}$) is calculated as follow^69^:

4 $$\begin{aligned} \Delta \mu _\text {O}(T, p_{\text {O}_2})&= \frac{1}{2}\left[ -k_\text {B}T \ln \left[ \left( \frac{2\pi m}{h^2}\right) ^\frac{3}{2}\left( k_\text {B}T\right) ^\frac{5}{2}\right] \right. \\&\quad + k_\text {B}T \ln p_{\text {O}_2} - k_\text {B}T \ln \left( \frac{8\pi ^2I_Ak_\text {B}T}{h^2}\right) \\&\quad + k_\text {B}T \ln \left[ 1-\exp \left( \frac{-h\nu _\text {OO}}{k_\text {B}T}\right) \right] \\&\quad \left. - k_\text {B}T \ln {\mathscr {M}} + k_\text {B}T \ln \sigma \right] , \end{aligned}$$

where *m* is the mass, $I_A$ is the moment of inertia of $\text {O}_2$ molecule, $\nu _\text {OO}$ is the O–O stretching frequency, ${\mathscr {M}}$ is the spin multiplicity and $\sigma$ is the symmetry number.

Under equilibrium growth condition, the chemical potentials are related to enthalpy of formation of $$\hbox {SrTiO}_3$$$$(\Delta \text {H}_\text {f}(\text {SrTiO}_3))$$ by:

5 $$\begin{aligned} \Delta \mu _\text {Sr} + \Delta \mu _\text {Ti} + 3\Delta \mu _\text {O} = \Delta \text {H}_\text {f}(\text {SrTiO}_3) \end{aligned}$$

Similarly, for $\hbox {TiO}_2$, the equilibrium growth condition is:

6 $$\begin{aligned} \Delta \mu _\text {Ti} + 2\Delta \mu _\text {O} = \Delta \text {H}_\text {f}(\text {TiO}_2) \end{aligned}$$

In addition, the chemical potentials are bounded by the following relation with enthalpy of formation to hinder the formation of secondary phases:

7 $$\begin{aligned}&\Delta \mu _{\text{Ti}} + 2\Delta \mu _{\text{O}} \le \Delta \text {H}_{\text{f}}(\text {TiO}_2)\\&\Delta \mu _{\text{Mn}} + 2\Delta \mu _{\text{O}} \le \Delta \text {H}_{\text{f}}(\text {MnO}_{2})\\&\Delta \mu _{\text{Ti}} + 2\Delta \mu _{\text{S}} \le \Delta \text {H}_{\text{f}}(\text {TiS}_{2})\\&\Delta \mu _{\text{Sr}} + \Delta \mu _{\text{O}} \le \Delta \text {H}_{\text{f}}(\text {SrO})\\&\Delta \mu _{\text{X}} \le 0; \quad (\text {X} = \text {Ti, Mn, Sr, O, S}) \end{aligned}$$

While solving, we have taken the equal sign in Eq. (7), except for the last bound. Therefore, we can see that the chemical potentials could be determined by imposing bounds on the formation of the precursors ($\hbox {MnO}_2$, $\hbox {TiO}_2$, $\hbox {TiS}_2$, $\hbox {Rh}_2\hbox {O}_3$) or the secondary phases and are inter-related.

**Figure 2:**
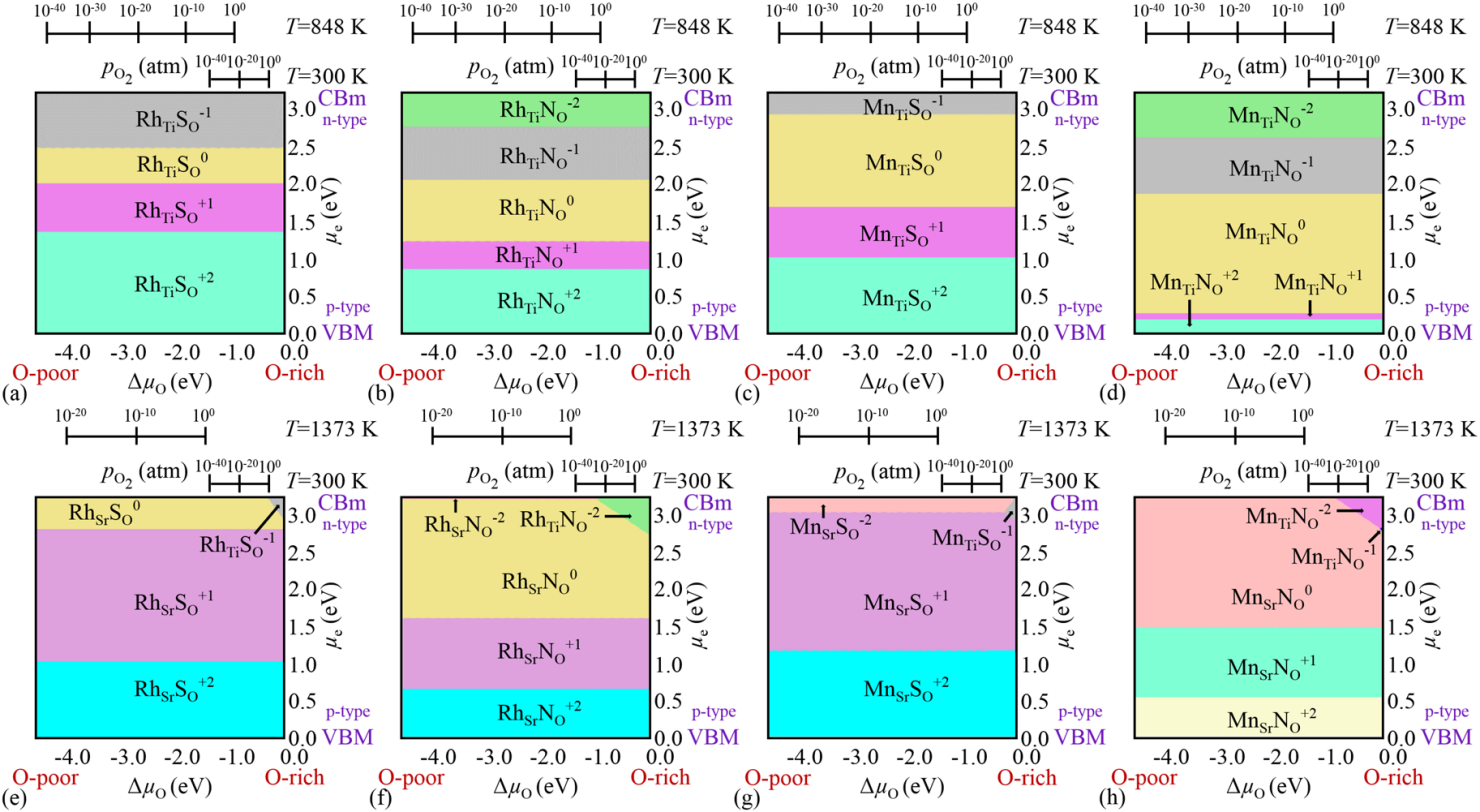
3D phase diagram that shows the most stable phases of (**a**) $\hbox {Rh}_\text {Ti}\hbox {S}_\text {O}$, (**b**) $\hbox {Rh}_\text {Ti}\hbox {N}_\text {O}$, (**c**) $\hbox {Mn}_\text {Ti}\hbox {S}_\text {O}$ and (**d**) $\hbox {Mn}_\text {Ti}\hbox {N}_\text {O}$ codoped $\hbox {TiO}_2$ charged configurations, and (**e**) S-Rh, (**f**) N-Rh, (**g**) S-Mn and (**h**) N-Mn^65^ codoped $\hbox {SrTiO}_3$ charged configurations with minimum formation energy as a function of $\Delta \mu _\text {O}$ and $\mu _\text {e}$. Here, $\Delta \mu _\text {O}$ (on x-axis) is varied according to environmental growth conditions [*T* and $p_{\text {O}_2}$ (on top axes)] and $\mu _\text {e}$ (on y-axis) is varied from VBM to CBm of pristine system. The experimental growth conditions for $\hbox {TiO}_2$ are $T = 848$ K, $p_{\text {O}_2} = 2.6\times 10^{-8}$ atm^73^ and for $\hbox {SrTiO}_3$ are *T* = 1373 K, $p_{\text {O}_2} = 1$ atm^37^.

We have taken three conditions (viz. O-rich, O-poor and O-intermediate) to calculate the formation energy of the Mn-S related defect in $\hbox {SrTiO}_3$ (see Fig. 1a–c). O-rich ($\Delta \mu _\text {O}=0$ eV) and O-poor ($\Delta \mu _\text {O}=-4.55$ eV) are the extreme growth conditions, which show the abundance and scarcity of O-content, respectively. For O-rich and O-poor conditions, $\Delta \mu _\text {O}$ is determined by the last bound of Eq. (7) and bound on the formation of $\hbox {TiO}_2$, respectively. O-intermediate condition ($\Delta \mu _\text {O}=-1.58$ eV) reflects the experimentally relevant condition (*T* = 1,373 K, $p_{\text {O}_2} = 1$ atm^37^) at which generally the growth of the system takes place. For O-intermediate condition, $\Delta \mu _\text {O}$ is calculated using Eq. (4). Under all the three conditions, ${\text {E}_\text {f}}({\text {Mn}_\text {Ti}}{\text {S}_\text {O}^{q}})$ depends only on the charge states *q*. Therefore, it remains same for all the three conditions. However, ${\text {E}_\text {f}}({\text {Mn}_\text {Sr}}{\text {S}_\text {O}^{q}})$ depends linearly on $\Delta \mu _\text {O}$ in addition to charge states *q*. Therefore, ${\text {E}_\text {f}}({\text {Mn}_\text {Sr}}{\text {S}_\text {O}^{q}})$ shifts by a constant amount for a particular growth condition. It is most negative in O-poor condition (see Fig. 1c). This implies that formation of ${{\text{{Mn}}}}_{{\text {{Sr}}}}{{\text {{S}}}}_{{\text {{O}}}}$ in $\hbox {SrTiO}_3$ is easier in O-poor condition. Moreover, from Fig. 1a–c, we can see that ${{\text {{Mn}}}}_{{\text {{Ti}}}}{{\text {{S}}}}_{{\text {{O}}}}$ is stable only in O-rich condition near CBm (n-type) with charge state $-1$, whereas, the positive charge states are stable for ${{\text{{Mn}}}}_{{\text {{Sr}}}}{{\text {{S}}}}_{{\text {{O}}}}$ codoped $\hbox {SrTiO}_3$. $+2$ charge state is stable near VBM and thereafter in between (+ 1) charge state is most stable. This indicates that the defect will act as a donor. Near CBm, $-2$ charge state is most stable. This whole result of 2D formation energy plot at different growth conditions can be summarized by a 3D phase diagram that shows only the most stable phases having minimum formation energy (see Fig. 2g). In 3D phase diagram, $\Delta \mu _\text {O}$ is varied according to environmental growth condition (on x-axis) and $\mu _\text {e}$ is varied throughout the band gap from VBM to CBm (on y-axis). On z-axis, we have shown the stable phases using colored surfaces, which have minimum formation energy. From Fig. 2g, we can easily see that ${{\text{{Mn}}}}_{{\text {{Sr}}}}{{\text {{S}}}}_{{\text {{O}}}}$ is stable in + 2, + 1 and − 2 charge states in all the conditions, which is also confirmed from Fig. 1. Whereas, ${{\text {{Mn}}}}_{{\text {{Ti}}}}{{\text {{S}}}}_{{\text {{O}}}}$ is only stable in − 1 charge state at O-rich condition near CBm, which can also be seen from Fig. 1a.

Figure 2 shows the stable phases of codoped $\hbox {TiO}_2$ and $\hbox {SrTiO}_3$ having minimum formation energy. Note that in case of codoped $\hbox {TiO}_2$, for different charge states, the dependence on chemical potential of oxygen is same for a particular kind of defect (for e.g. Mn substituted at Ti and S substituted at O). Since we have shown only one kind of defect i.e., only substitution at particular sites (a metal at Ti and a nonmetal at O) with different charge states, the formation energy looks independent of chemical potential of O (see Fig. 2a–d). Whereas, in case of codoped $\hbox {SrTiO}_3$, there are two types of defects in which a metal can be substituted either at Ti or Sr position and a nonmetal at O-site as shown in 3D phase diagram (see Fig. 2e–h). These stable phases include charged defects in addition to neutral defects due to the uncompensated charge. The positive charge states are stable near VBM (in p-type host), whereas the negative charge states are stable near CBm (in n-type host). The $\hbox {Rh}_\text {Ti}\hbox {S}_\text {O}$ and $\hbox {Mn}_\text {Ti}\hbox {S}_\text {O}$ codoped $\hbox {TiO}_2$ could be stable in + 2, + 1, 0, and − 1 charge states (see Fig. 2a,c). These codopants will act as donor in p-type host (near VBM), and acceptor in n-type host (near CBm). The charge state − 2 is not stable in both the cases. As these transition metals have partially filled d-orbitals, it could accept electrons from the host as well as donate electrons to the host. Figure 2b,d show that $\hbox {Rh}_\text {Ti}\hbox {N}_\text {O}$ and $\hbox {Mn}_\text {Ti}\hbox {N}_\text {O}$ codoped $\hbox {TiO}_2$ will be stable in − 2 charge state in addition to + 2, + 1, 0 and − 1, since N has one electron less than O, it could accept one electron extra in comparison to S (S has same number of valence electrons as for O). In $\hbox {Mn}_\text {Ti}\hbox {S}_\text {O}$ and $\hbox {Mn}_\text {Ti}\hbox {N}_\text {O}$ codoped $\hbox {TiO}_2$, for a large range of $\mu _\text {e}$, neutral charge state is more favorable. The Sr site is more favorable than Ti site for substitution in $\hbox {SrTiO}_3$ (see Fig. 2e–h). The Ti site could be substituted in O-rich (Ti-poor) condition near CBm (in n-type host). Mostly, $\hbox {Rh}_\text {Sr}\hbox {S}_\text {O}$, $\hbox {Rh}_\text {Sr}\hbox {N}_\text {O}$, $\hbox {Mn}_\text {Sr}\hbox {S}_\text {O}$, and $\hbox {Mn}_\text {Sr}\hbox {N}_\text {O}$ codopants act as donor as they are stable in + 2 and + 1 charge states for a wide range of $\mu _\text {e}$ or in neutral charge state. However, when metal is substituted at the Ti position, the defect configuration will act as an acceptor. Similar to the codoped $\hbox {TiO}_2$, the $\hbox {Rh}_\text {Ti}\hbox {N}_\text {O}$, and $\hbox {Mn}_\text {Ti}\hbox {N}_\text {O}$ codoped $\hbox {SrTiO}_3$ will get stabilized after accepting one extra electron in comparison to $\hbox {Rh}_\text {Ti}\hbox {S}_\text {O}$, and $\hbox {Mn}_\text {Ti}\hbox {S}_\text {O}$.

### Electronic density of states (DOS)

**Figure 3:**
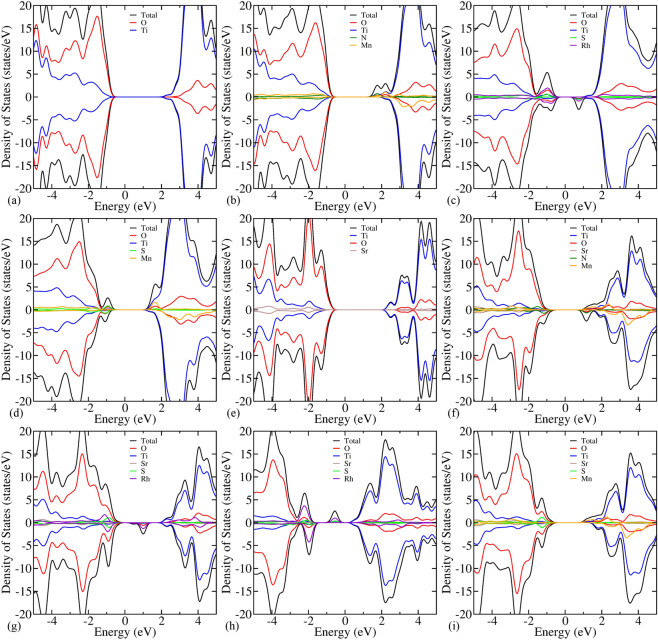
Atom projected density of states of (**a**) pristine $\hbox {TiO}_2$, (**b**) $\hbox {Mn}_\text {Ti}\hbox {N}_\text {O}$, (**c**) $\hbox {Rh}_\text {Ti}\hbox {S}_\text {O}$, (**d**) $\hbox {Mn}_\text {Ti}\hbox {S}_\text {O}$ codoped $\hbox {TiO}_2$, and (**e**) pristine $\hbox {SrTiO}_3$, (**f**) ${{\hbox {{Mn}}}}_{{\text {{Ti}}}}{{\text {{N}}}}_{{\text {{O}}}}$^65^, (**g**) ${{\hbox {{Rh}}}}_{{\text {{Ti}}}}{{\text {{S}}}}_{{\text {{O}}}}$, (**h**) $\hbox {Rh}_\text {Sr}\hbox {S}_\text {O}$, and (**i**) $\hbox {Mn}_\text {Ti}\hbox {S}_\text {O}$ codoped $\hbox {SrTiO}_3$.

The defect states could be seen by means of electronic density of states. Figure 3 shows the atom projected density of states for pristine and codoped $\hbox {TiO}_2$ as well as $\hbox {SrTiO}_3$. In the pristine systems, near Fermi level, the valence band is contributed by O 2p orbitals and the conduction band is contributed by Ti 3d orbitals (see Fig. 3a,e). The DOS is symmetric w.r.t. the spin alignments ascribed to the paired electrons in the system. On doping, the DOS becomes asymmetric attributable to unpaired electrons. These unpaired electrons tend to result in finite magnetic moment. The details of magnetic moment for codoped systems are given in Supplementary Tables S1 and S2 (see Section I of SI). The deep trap states arise in $\hbox {N}_\text {O}$, $\hbox {Rh}_\text {Ti}$ and $\hbox {Rh}_\text {Ti}\hbox {N}_\text {O}$ doped $\hbox {TiO}_2$, that increase the recombination rate and deteriorate the photocatalytic efficiency (see Supplementary Fig. S1a,d,e). There is very slight reduction in band gap for $\hbox {Mn}_\text {Ti}$ doped $\hbox {TiO}_2$. Hence, it’s not inducing the effective visible light absorption (see Supplementary Fig. S1c). In $\hbox {Mn}_\text {Ti}\hbox {N}_\text {O}$ codoped $\hbox {TiO}_2$, the N orbitals and Mn orbitals shift down the CBm, leading to the reduction in band gap and induce the visible light absorption (see Fig. 3b). However, this shift will lower down the CBm and thus, not efficient for reduction of water to produce hydrogen. The VBM is elevated in $\hbox {Rh}_\text {Ti}\hbox {S}_\text {O}$ codoped $\hbox {TiO}_2$, which is caused by the S and Rh orbitals contribution (see Fig. 3c). Furthermore, the CBm is also shifted down due to the unoccupied states of S and Rh orbitals. Therefore, despite its spectral response in visible region, it cannot be used for producing oxygen via water reduction ascribed to the large shift in VBM. The band gap reduction is induced by the S orbitals in $\hbox {S}_\text {O}$ doped $\hbox {TiO}_2$ as the S orbitals energies lie higher than the N orbitals (see Supplementary Fig. S1b). Also, in $\hbox {Mn}_\text {Ti}\hbox {S}_\text {O}$ codoped $\hbox {TiO}_2$, the S orbitals elevate the VBM as they have higher energy than the O orbitals and the Mn orbitals contribute to CBm (see Fig. 3d). This will enhance the photocatalytic efficiency, as the band gap becomes 2.2 eV in both the aforementioned cases and the band edges straddle the redox potentials of water. Note that the energy gap is defined as band gap between the highest occupied and lowest unoccupied band, provided that defect level is not far away from VBM and CBm. If it forms continuous states with VBM/CBm, i.e. if the defect level is shallower, then only, we have claimed it as the VBM/CBm. If the defect is localized in between the forbidden region (band gap), then we do not consider that state as either VBM or CBm.

For $\hbox {SrTiO}_3$, $\hbox {N}_\text {O}$ and $\hbox {S}_\text {O}$ behave similar to the case of $\hbox {N}_\text {O}$ and $\hbox {S}_\text {O}$ monodoped $\hbox {TiO}_2$ (see Supplementary Fig. S1f,g). However, the reduction in band gap for $\hbox {S}_\text {O}$ is small in comparison to $\hbox {S}_\text {O}$ doped $\hbox {TiO}_2$. $\hbox {S}_\text {O}$ doped $\hbox {SrTiO}_3$ has the band gap of 2.59 eV and hence, responses to visible light irradiation. For $\hbox {Mn}_\text {Ti}$ monodoped $\hbox {SrTiO}_3$, the Mn orbital contributes to the CBm and lowers down it (see Supplementary Fig. S1h). Therefore, its spectral response expands to visible light irradiation (band gap is 2.57 eV). In $\hbox {Rh}_\text {Ti}$ monodoped $\hbox {SrTiO}_3$, the unoccupied states of Rh orbitals appear at VBM, and the difference between highest occupied and lowest unoccupied state is 0.23 eV (see Supplementary Fig. S1i). Thus, it is not a promising candidate for enhanced photocatalytic activity. The lowering of CBm in $\hbox {Rh}_\text {Sr}$ is occured due to the Rh localized states contribution to CBm and therefore, it doesn’t have enough reduction power to produce hydrogen via water splitting (see Supplementary Fig. S1j). There is no reduction in band gap for $\hbox {Mn}_\text {Sr}$ monodoped $\hbox {SrTiO}_3$, as the Mn orbitals contribute deep inside the valence and conduction band (see Supplementary Fig. S1k). Likewise $\hbox {Mn}_\text {Ti}\hbox {N}_\text {O}$ codoped $\hbox {TiO}_2$, the reduction in band gap of $\hbox {Mn}_\text {Ti}\hbox {N}_\text {O}$ codoped $\hbox {SrTiO}_3$ is occurred by lowering of the CBm as well as elevation of the VBM (see Fig. 3f). However, the shift in CBm is large enough such that its reduction power is deteriorated. In $\hbox {Rh}_\text {Ti}\hbox {S}_\text {O}$ and $\hbox {Rh}_\text {Sr}\hbox {S}_\text {O}$ codoped $\hbox {SrTiO}_3$, the deep trap states arise in the forbidden region, that increase the recombination of photogenerated charge carriers and thus, degrade the photocatalytic activity (see Fig. 3g,h). Since there is occurrence of trap states in $\hbox {Rh}_\text {Ti}\hbox {N}_\text {O}$ and $\hbox {Mn}_\text {Sr}\hbox {S}_\text {O}$ codoped $\hbox {SrTiO}_3$, it will result in poor photocatalytic activity (see Supplementary Fig. S1l,o). The Rh and N orbitals states elevate the VBm of $\hbox {Rh}_\text {Sr}\hbox {N}_\text {O}$ codoped $\hbox {SrTiO}_3$, which results in a band gap of 2.69 eV (see Supplementary Fig. S1m). The $\hbox {Mn}_\text {Ti}\hbox {S}_\text {O}$ codoped $\hbox {SrTiO}_3$ has the band gap of 1.95 eV. The VBM elevation is concomitant with occurrence of S orbitals at VBM and the Mn orbitals at CBm (see Fig. 3i). The shifts in CBm and VBM are such that the band edges straddle the redox potential levels of water. Similarly, for $\hbox {Mn}_\text {Sr}\hbox {N}_\text {O}$ codoped $\hbox {SrTiO}_3$, the band gap is 2.17 eV, and the defect states are shallower, which serves the purpose of efficient photocatalyst (see Supplementary Fig. S1n).

### Optical properties

**Figure 4:**
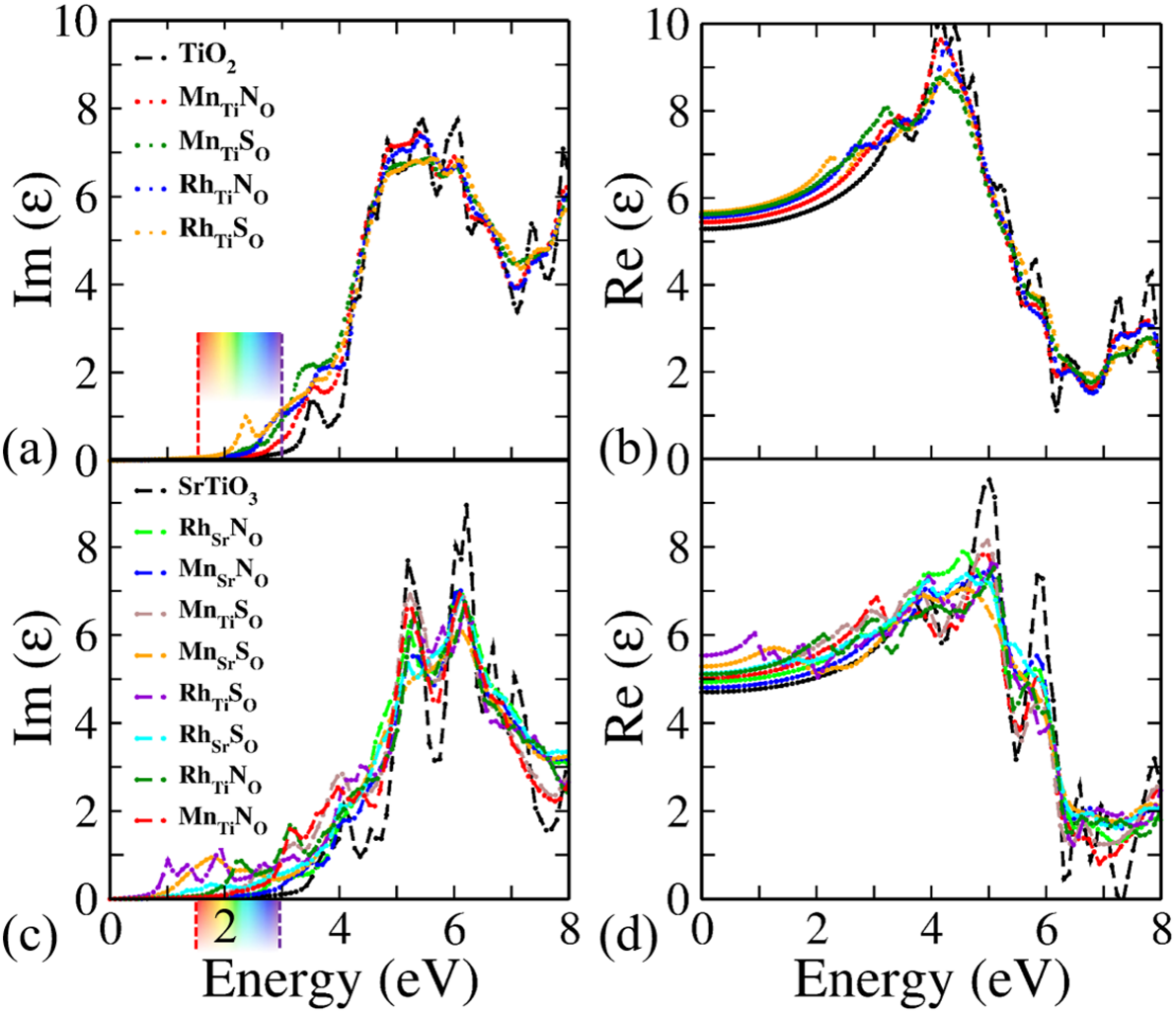
Spatially average (**a**) imaginary (Im $\varepsilon$) and (**b**) real (Re $\varepsilon$) part of the dielectric function for (un)doped $\hbox {TiO}_2$, (**c**) imaginary (Im $\varepsilon$) and (**d**) real (Re $\varepsilon$) part for (un)doped $\hbox {SrTiO}_3$.

The optical spectra have been determined by calculating the frequency dependent complex dielectric function $\varepsilon (\omega ) = \text {Re} (\varepsilon ) + \text {Im}(\varepsilon )$ using HSE06 functional. The real part $\text {Re}(\varepsilon )$ and the imaginary part $\text {Im}(\varepsilon )$ are associated with the electronic polarizability and optical absorption of the material, respectively. The sum of all possible transitions from the occupied to the unoccupied states gives the direct interband transition, which is reflected in the imaginary part of the dielectric function. The imaginary and real part for codoped anatase $\hbox {TiO}_2$ and $\hbox {SrTiO}_3$ are shown in Fig. 4 (the results for monodoped $\hbox {TiO}_2$ and $\hbox {SrTiO}_3$ are shown in Supplementary Fig. S2). Note that anatase $\hbox {TiO}_2$ has tetragonal structure. Therefore, the optical anisotropy is also associated with it. The detailed discussion of optical anisotropy is already done in our previous work^74^. Therefore, here we have shown only the averaged (x, y, z polarizations) for imaginary and real part of the dielectric function. The imaginary part of dielectric function shows the first peak at 3.56 eV for pristine $\hbox {TiO}_2$ as shown in Fig. 4a (matching with the previous works, which is 3.8 eV^75^). The peaks are shifted to lower energy for codoped cases. This enhances the visible light absorption of anatase $\hbox {TiO}_2$. The static real part of the dielectric function (at $\omega = 0$) for $\hbox {TiO}_2$ is found to be 5.28 (see Fig. 4b), which is very close to the experimental value i.e., 5.62^76^. On codoping its value is increased.

For the case of cubic $\hbox {SrTiO}_3$, the spatially average imaginary and real part of dielectric function are shown in Fig. 4c,d, respectively. The static real part of the dielectric function for pristine $\hbox {SrTiO}_3$ is estimated as 4.7 (experimental value is 5.27^77^) and its value is increased with codopants (see Fig. 4d). The first absorption peak is observed at 4.08 eV for pristine $\hbox {SrTiO}_3$ as shown in Fig. 4c (experimental value is 4.7 eV^77^). Likewise in anatase $\hbox {TiO}_2$, the peaks are shifted to visible region for the codoped cases. Note that the optical properties in the high energy range are controlled by the electronic transitions between O 2p states and Ti 3d states. Therefore, the spectra of all the configurations are nearly identical in high energy range. However, the optical properties in low energy range (less than 3 eV) are different, these are affected by the transitions involving the impurity states. The observed visible light absorption could be ascribed to the presence of the dopant states (as shown in DOS near Fermi-level), which reduce the electron transition gap for optical absorption. This leads to a new absorption edge in the visible light region.

### Band edge alignment

**Figure 5:**
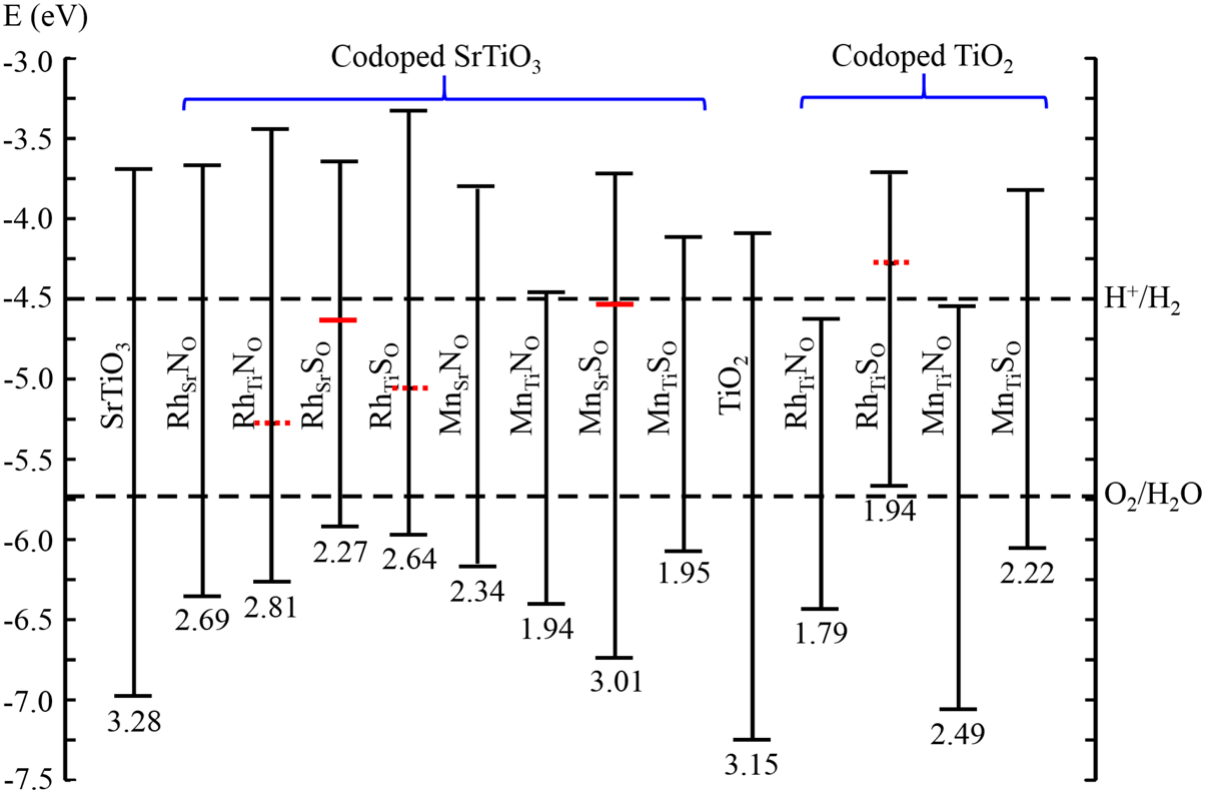
Band edge alignment of (un)doped $\hbox {SrTiO}_3$ and $\hbox {TiO}_2$ w.r.t. water reduction and oxidation potential levels (H$^+$/$\hbox {H}_2$, $\hbox {O}_2$/$\hbox {H}_2$O). The solid and dashed red line in forbidden region are representing the highest occupied and lowest unoccupied states, respectively.

The band edge alignment has been performed to obtain the potential candidates for photocatalytic water splitting. The CBm should lie above water reduction potential and VBM should lie below water oxidation potential for overall water splitting. Note that we have adopted the standard methodology to align the band edges as in Refs.^78,79^. First we align the band edges of pristine $\hbox {TiO}_2$ and $\hbox {SrTiO}_3$ w.r.t. water redox potential. For pristine $\hbox {SrTiO}_3$, the CBm lies 0.8 eV above the water reduction potential and VBM lies 1.25 eV below water oxidation potential, which is reported in the experimental study^49^. Further, we align the band edges of defected configurations by observing the shift in CBm and VBM w.r.t. the pristine system (see Fig. 5). Similarly, we have aligned the band edges of (un)doped $\hbox {TiO}_2$. For pristine $\hbox {TiO}_2$, the CBm lies 0.4 eV above the water reduction potential^48^. The band edge alignment for monodoped anatase $\hbox {TiO}_2$ and $\hbox {SrTiO}_3$ are shown in Supplementary Fig. S3. The monodoped $\hbox {N}_\text {O}$ is not suitable in both the cases (anatase $\hbox {TiO}_2$ and $\hbox {SrTiO}_3$), as it results in deep trap states. This increases the recombination and decreases the mobility of photogenerated charge carriers (see Supplementary Fig. S3). Likewise, for Rh dopant (in monodoping as well as in codoping), there is occurrence of trap states. These states degrade the photocatalytic efficiency. Therefore, mono- and codopoing of Rh with a nonmetal could reduce the band gap, but it cannot be an efficient photocatalyst in $\hbox {TiO}_2$ as well as in $\hbox {SrTiO}_3$.

The monodoped $\hbox {S}_\text {O}$ in both anatase $\hbox {TiO}_2$ as well as $\hbox {SrTiO}_3$ could enhance the photocatalytic efficiency and split water as their band edges straddle the redox potential of water (see Supplementary Fig. S3). However, in $\hbox {S}_\text {O}$ monodoped $\hbox {SrTiO}_3$, the band gap (2.59) is slightly higher than the desirable band gap ($\sim$ 2 eV^51,52^), and thus, its efficiency will be smaller. Similarly, for $\hbox {Mn}_\text {Ti}$ monodoped $\hbox {SrTiO}_3$, the band gap is 2.57 eV, and due to shift of its CBm towards Fermi level, its reduction power will be degraded (see Fig. S3). On the other hand, for $\hbox {Mn}_\text {Sr}$ monodoped $\hbox {SrTiO}_3$, and $\hbox {Mn}_\text {Ti}$ monodoped anatase $\hbox {TiO}_2$, the slight change in band gap is observed and thus, these can not enhance the photocatalytic activity.

In $\hbox {Rh}_\text {Ti}\hbox {S}_\text {O}$ codoped $\hbox {TiO}_2$, since there is manifestation of deep unoccupied states as well as the VBM lies above the oxidation potential of water, it could not be utilized for photocatalytic overall water splitting (see Fig. 5). For $\hbox {Rh}_\text {Ti}\hbox {N}_\text {O}$ and $\hbox {Mn}_\text {Ti}\hbox {N}_\text {O}$ codoped $\hbox {TiO}_2$, the CBm lies below the reduction potential of water, and thus, cannot produce hydrogen via water splitting. In $\hbox {TiO}_2$, only the $\hbox {Mn}_\text {Ti}\hbox {S}_\text {O}$ codoping is the potential candidate for overall photocatalytic water splitting as it has a desirable band gap of 2.22 eV and it does not contain the trap states in forbidden region while retaining the sufficient reduction and oxidation power for hydrogen evolution reaction (HER) as well as oxygen evolution reaction (OER). Similarly, in $\hbox {SrTiO}_3$, except $\hbox {Rh}_\text {Sr}\hbox {N}_\text {O}$, the Rh doping does not aid in enhancing the photocatalytic activity ascribed to the formation of recombination centers. $\hbox {Rh}_\text {Sr}\hbox {N}_\text {O}$ defect configuration enhances the photocatalytic efficiency, however its band gap (2.69 eV) is little bit larger in comparison to the maximum efficient photocatalyst ($\sim$ 2 eV). In $\hbox {Mn}_\text {Sr}\hbox {S}_\text {O}$, since the occupied deep states lie below the CBm and also these are not the shallow impurity levels, this configuration is not a desirable photocatalyst. The reduction in band gap for $\hbox {Mn}_\text {Ti}\hbox {N}_\text {O}$ is concomitant with the lowering of CBm, that deteriorates its reduction power. The $\hbox {Mn}_\text {Sr}\hbox {N}_\text {O}$, and $\hbox {Mn}_\text {Ti}\hbox {S}_\text {O}$ codoped $\hbox {SrTiO}_3$ configurations are the potential candidates for overall photocatalytic water splitting attributable to their desirable band gap ($\sim$ 2 eV) with congenial band edge positions.

### Band structure and effective mass

To see the effect on mobility due to the defects, we have calculated the effective mass of charge carriers (using HSE06) of those systems, which could be promising candidates for overall photocatalytic water splitting (see Table 1).

**Table 1:** Effective masses (in terms of free-electron mass $\hbox {m}_\text {e}$) at the band edges. The masses $\text {m}_\text {he}, \text {m}_\text {le},\text {m}_\text {hh}, \text {and}\;\text {m}_\text {lh}$ correspond to heavy-electron, light-electron, heavy-hole, and light-hole band, respectively.

Systems	$\text {m}_\text {he}$	$\text {m}_\text {le}$	$\text {m}_\text {hh}$	$\text {m}_\text {lh}$
Pristine $\hbox {SrTiO}_3$	5.18	0.38	$-10.36$	$-0.74$
$\hbox {Mn}_\text {Sr}\hbox {N}_\text {O}$ codoped $\hbox {SrTiO}_3$	3.04	–	–	$-1.53$
$\hbox {Mn}_\text {Ti}\hbox {S}_\text {O}$ codoped $\hbox {SrTiO}_3$	–	0.25	–	$-0.66$
pristine $\hbox {TiO}_2$	–	0.39	$-1.57$	–
$\hbox {Mn}_\text {Ti}\hbox {S}_\text {O}$ codoped $\hbox {TiO}_2$	–	0.45	$-9.23$	–
$\hbox {S}_\text {O}$ monodoped $\hbox {TiO}_2$	–	0.41	$-2.84$	–

**Figure 6:**
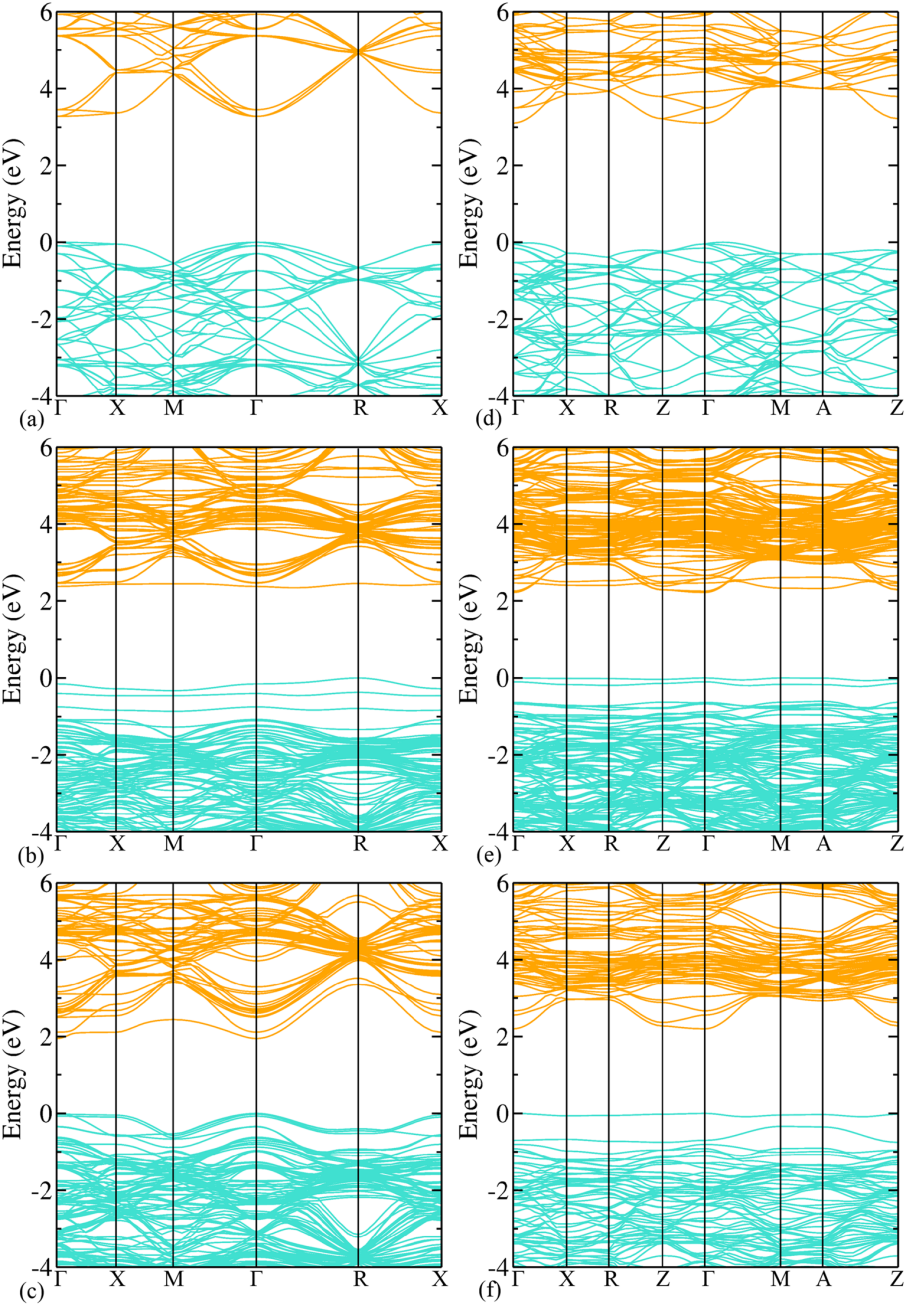
Band structure calculated using hybrid (HSE06) functional of (**a**) pristine, (**b**) $\hbox {Mn}_\text {Sr}\hbox {N}_\text {O}$ codoped^65^, (**c**) $\hbox {Mn}_\text {Ti}\hbox {S}_\text {O}$ codoped $\hbox {SrTiO}_3$ and (**d**) pristine, (**e**) $\hbox {Mn}_\text {Ti}\hbox {S}_\text {O}$ codoped, (**f**) $\hbox {S}_\text {O}$ monodoped $\hbox {TiO}_2$.

These are obtained from the relation of effective mass ($m^*$) with second derivative of energy with respect to *k* (wave vector) at the band edges:

8 $$\begin{aligned} \frac{1}{m^*} = \frac{1}{{\hslash }^2}\frac{d^2E}{dk^2}\text {,} \end{aligned}$$

where $\hslash$ is the reduced Planck constant. The effective masses of charge carriers of pristine $\hbox {SrTiO}_3$ are validated with Refs.^79–81^. Except for the heavy hole, all are matching well. For pristine $\hbox {SrTiO}_3$, the effective masses are calculated along $\Gamma$-X high symmetry path. Pristine has degenerate bands at the $\Gamma$ k-point (see Fig. 6a). In contrast to pristine $\hbox {SrTiO}_3$, rest of the cases have non-degenerate bands (highest occupied and lowest unoccupied) (see Fig. 6). Note that here, we have shown the total bands containing both the spin up and spin down contribution. For individual contribution, we have shown the spin up and spin down bands for two test cases in Section V of SI (see Supplementary Figs. S4, S5). The electron’s effective mass of $\hbox {Mn}_\text {Sr}\hbox {N}_\text {O}$ codoped $\hbox {SrTiO}_3$ is 3.04$\hbox {m}_\text {e}$ and 5.09$\hbox {m}_\text {e}$ along CBm-X and CBm-$\Gamma$ path, respectively, and the hole’s effective mass is $1.53\hbox{m}_\text {e}$ and − 2.58$\hbox {m}_\text {e}$ along R-X and R-$\Gamma$, respectively. These different values along different directions indicate the anisotropic nature of effective mass. For $\hbox {Mn}_\text {Ti}\hbox {S}_\text {O}$ codoped $\hbox {SrTiO}_3$, the effective mass of both the charge carriers (calculated along $\Gamma$-X direction) is decreased. It is also clear from the large curvature of the bands around CBm and VBM in comparison to pristine $\hbox {SrTiO}_3$ (see Fig. 6c).

In pristine $\hbox {TiO}_2$ the CBm is at $\Gamma$ k-point and there is no degeneracy (see Fig. 6d). The electron’s effective mass is 0.39$\hbox {m}_\text {e}$ along $\Gamma$-Z high-symmetry path, and the effective mass of hole is $-1.57\hbox {m}_\text {e}$ along VBM-Z and $-1.61\hbox {m}_\text {e}$ along VBM-$\Gamma$ direction. For $\hbox {Mn}_\text {Ti}\hbox {S}_\text {O}$ codoped $\hbox {TiO}_2$ and $\hbox {S}_\text {O}$ monodoped $\hbox {TiO}_2$, the electron’s effective mass (along $\Gamma$-Z) is comparable with pristine, whereas the hole’s effective mass (along VBM-Z) is increased. These increments are also evident from the smaller curvature of the bands around the band edges (see Fig. 6e,f). For larger effective mass, the mobility will be smaller and the recombination rate will also be greater. Therefore, from Table 1, we can see that in case of $\hbox {Mn}_\text {Ti}\hbox {S}_\text {O}$ codoped $\hbox {SrTiO}_3$, the mobility of charge carriers will be large, and for rest of the cases, the effective mass values are comparable and the mobility will not be affected much. This is because, the mobility depends on both the effective mass and scattering (relaxation) time. On doping, the scattering rate is expected to get decreased as the degeneracy will be lifted. As a consequence of this, despite of small increment in effective mass, the mobility will not be affected considerably, especially here due to low doping concentration^82^. These effective mass studies should assist future experimental as well as theoretical investigations to tailor the transport properties of the system.

## Conclusions

In summary, we have evaluated the thermodynamic stability of (un)doped anatase $\hbox {TiO}_2$ and $\hbox {SrTiO}_3$ using hybrid DFT and ab initio atomistic thermodynamics. We have found that the codopants in $\hbox {TiO}_2$ could act as donor (in p-type host) as well as acceptor (in n-type host). However, the most stable codopants (codoping of metal at Sr site and nonmetal at O site) in $\hbox {SrTiO}_3$ mostly act as donors. The codoping expands the spectral response and induces visible light in both the cases. However, the recombination centers are present in Rh-related defect configurations attributable to Rh localized orbitals in the forbidden region and moreover, there is a large shift in the CBm or VBM. This will lead to degradation in photocatalytic efficiency. The mobility of charge carriers is maximum in $\hbox {Mn}_\text {Ti}\hbox {S}_\text {O}$ codoped $\hbox {SrTiO}_3$, and in rest of the cases, it is not affected much. Our results reveal that $\hbox {Mn}_\text {Ti}\hbox {S}_\text {O}$ codoped, $\hbox {S}_\text {O}$ monodoped anatase $\hbox {TiO}_2$, $\hbox {Mn}_\text {Ti}\hbox {S}_\text {O}$ and $\hbox {Mn}_\text {Sr}\hbox {N}_\text {O}$ codoped $\hbox {SrTiO}_3$ are the most favorable candidates for enhancing photocatalytic overall water splitting owing to the passivation of trap states and congenial band edge positions with desirable visible light absorption.

## Methods

We have performed the DFT calculations as implemented in Vienna ab initio simulation package (VASP)^83,84^. The projector-augmented wave (PAW) pseudopotentials^85^ have been used to describe the interactions between electrons and ions for all the species. For the energy calculations, hybrid exchange-correlation (xc) functional HSE06^86^ is used. Note that we have seen in our previous study that GGA+U is not a good functional to predict the correct energetics, albeit it can reproduce the correct band gap with suitable value of U^64^. The exact exchange fractions in HSE06 functional used for $\hbox {TiO}_2$ and $\hbox {SrTiO}_3$ are 22% and 28%, respectively (see SI Ref.^64,65^ for validation of exact exchange fraction). The band gap of 3.15 eV and 3.28 eV are reproduced for $\hbox {TiO}_2$ and $\hbox {SrTiO}_3$ respectively, which are well in agreement with the experimental values^87,88^. To make the defect to be localized, we have used $2\times 2\times 1$ (48-atom) and $2\times 2\times 2$ (40-atom) supercells by replication of $\hbox {TiO}_2$ and $\hbox {SrTiO}_3$ unit cells respectively (for validation of supercell size, see section VI of SI and also, the localized states due to defects can be seen from band structure in Fig. 6). The k-grid for Brillouin zone sampling is generated using Monkhorst–Pack^89^ scheme and all results are checked for convergence w.r.t. the mesh size ($4\times 4\times 4$). The electronic self-consistency loop for the total energy is converged with a threshold of 0.01 meV. An energy cutoff of 600 eV is used for the plane wave basis set. Note that the spin-polarized calculations have been carried out since the doped systems contain unpaired electrons.

## Supplementary information

Supplementary material 1
