# Supplementary material for: Theoretical insights of codoping to modulate electronic structure of TiO2 and SrTiO3 for enhanced photocatalytic efficiency
Source: Sci Rep. 2020 Sep 21;10:15372. doi: 10.1038/s41598-020-72195-0 (PMC7505848; doi:10.1038/s41598-020-72195-0)
Supplement: Supplementary file 1 — Supplementary material 1 [file 41598_2020_72195_MOESM1_ESM.pdf]

Theoretical insights of codoping to modulate electronic structure of TiO_2 and $SrTiO_3$ for enhanced photocatalytic efficiency

Manish Kumar*, Pooja Basera, Shikha Saini, Saswata Bhattacharya*
Department of Physics, Indian Institute of Technology Delhi, New Delhi 110016 India
*Email: manish.kumar@physics.iitd.ac.in [MK], saswata@physics.iitd.ac.in [SB]

Supplemental Material

I. Magnetic moment of codoped anatase TiO_2 and $SrTiO_3$

II. Electronic density of states (DOS) of doped anatase TiO_2 and $SrTiO_3$

III. Optical properties of monodoped anatase TiO_2 and $SrTiO_3$

IV. Band edge alignment of monodoped anatase TiO_2 and $SrTiO_3$

V. Spin up and spin down bands of $Mn_{Ti}S_O$ codoped anatase TiO_2 and $Mn_{Sr}N_O$ codoped $SrTiO_3$

VI. Validation of supercell size of anatase TiO_2

I. Magnetic moment of codoped anatase TiO_2 and $SrTiO_3$

TABLE S1: Magnetic moment of codoped TiO_2 systems

Codopants	magnetic moment
$Mn_{Ti}S_O$	3.00
$Mn_{Ti}N_O$	2.00
$Rh_{Ti}S_O$	1.00
$Rh_{Ti}N_O$	0.00

TABLE S2: Magnetic moment of codoped $SrTiO_3$ systems

Codopants	magnetic moment
$Mn_{Ti}S_O$	3.00
$Mn_{Ti}N_O$	4.00
$Rh_{Ti}S_O$	1.00
$Rh_{Ti}N_O$	2.00
$Mn_{Sr}S_O$	-5.00
$Mn_{Sr}N_O$	4.00
$Rh_{Sr}S_O$	1.00
$Rh_{Sr}N_O$	0.00

The magnetic moment depends on the unpaired electrons in the system. Therefore, due to different oxidation states of Mn at different sites, as well as due to different number of unpaired electrons in different codoped systems, the magnetic moment is different. In case of TiO_2, for $Mn_{Ti}S_O$ codoping, the Mn is in 4+ oxidation state similar to Ti^{4+}. Therefore, the magnetic moment for the system is $3\mu_B$, since Mn d-orbitals have 3 unpaired electrons left. Whereas, for $Mn_{Ti}N_O$ codoping, the N has one electron less than the O, therefore, the unpaired electron in the system will be 2 (of Mn d-orbitals, since one of the electrons of d-orbitals is paired with an electron of p-orbitals of N). It leads to magnetic moment of $2\mu_B$.

Similarly, in the case of $SrTiO_3$, for $Mn_{Ti}S_O$ codoping, the magnetic moment is $3\mu_B$. However, for $Mn_{Ti}N_O$ codoping, the magnetic moment is $4\mu_B$. This is due to the fact that it has 4 unpaired electrons. In this case, the Mn and N are not directly linked as in the case of TiO_2. Therefore, it has 3 unpaired electrons from Mn d-orbitals and 1 unpaired electron from N p-orbitals (the Mn d-orbitals and N p-orbitals are not hybridized).

For $Mn_{Sr}S_O$ codoping in $SrTiO_3$, the Mn is in 2+ oxidation state similar to Sr^{2+}. Therefore, the magnetic moment for the system is -$5\mu_B$, since Mn d-orbitals have 5 unpaired electrons left. Whereas, for $Mn_{Sr}N_O$ codoping, the N has one electron less than the O, therefore, the unpaired electron in the system will be 4 (of Mn d-orbitals), since an electron of d-orbitals of Mn will be paired with one electron of p-orbitals of N (here, Mn and N are linked directly). It leads to magnetic moment of $4\mu_B$ (The sign is simply due to spin up or spin down of each unpaired electron).

Similarly, we can find the magnetic moment in Rh-N, Rh-S codoped TiO_2 and $SrTiO_3$.

II. Electronic density of states (DOS) of doped anatase TiO_2 and $SrTiO_3$

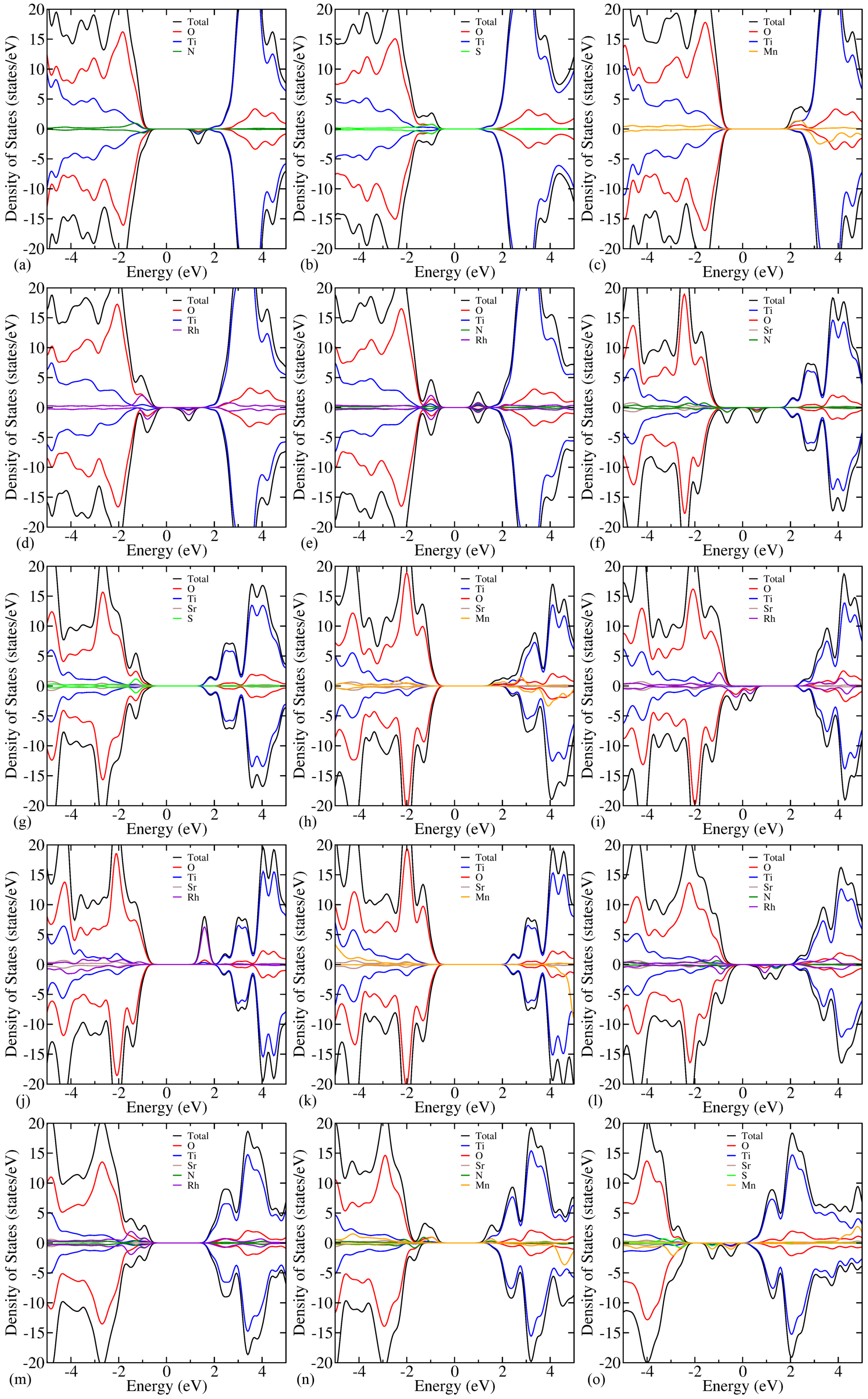

Figure S1: Atom projected density of states of (a) N_O, (b) S_O, (c) Mn_{Ti}, (d) Rh_{Ti} (e) $Rh_{Ti}N_O$ codoped TiO_2, and (f) N_O, (g) S_O, (h) Mn_{Ti}, (i) Rh_{Ti}, (j) Rh_{Sr}, (k) Mn_{Sr}, (l) $Rh_{Ti}N_O$, (m) $Rh_{Sr}N_O$, (n) $Mn_{Sr}N_O$ and (o) $Mn_{Sr}S_O$ codoped $SrTiO_3$.

III. Optical properties of monodoped anatase TiO_2 and $SrTiO_3$

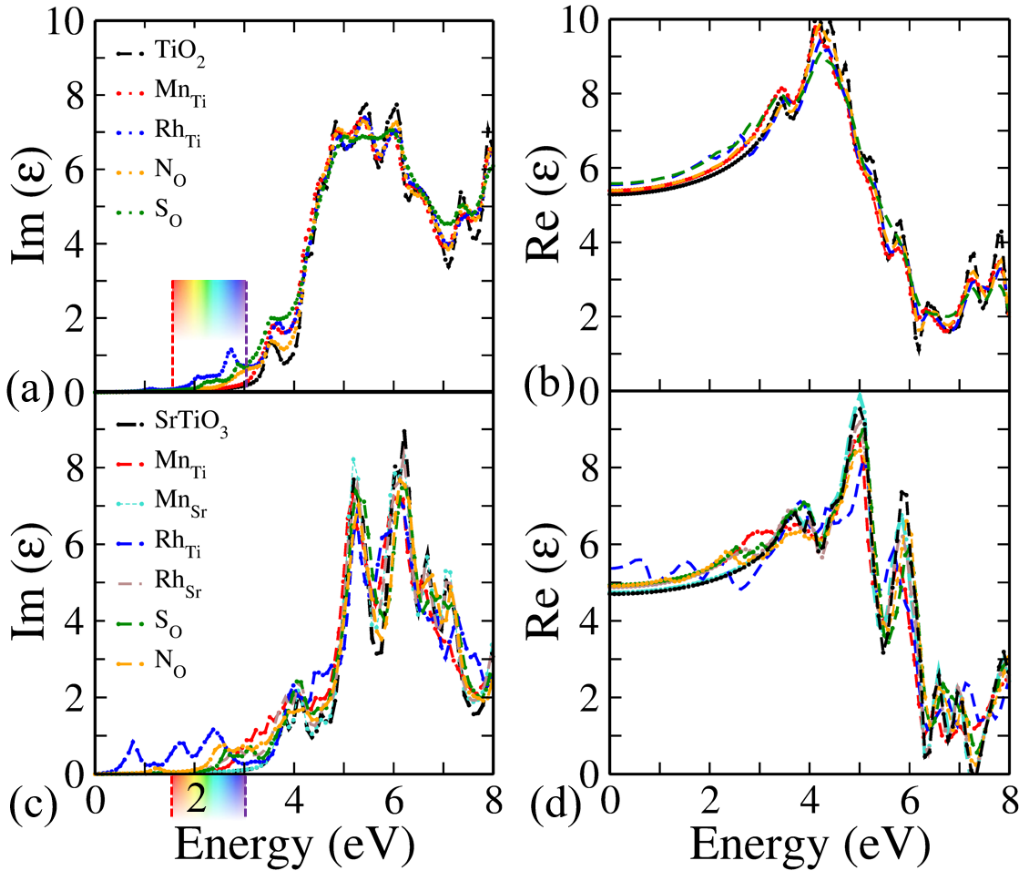

Figure S2: Spatially average (a) imaginary (Im ε) and (b) real (Re ε) part of the dielectric function for (un)doped TiO_2, (c) imaginary (Im ε) and (d) real (Re ε) part for (un)doped $SrTiO_3$.

IV. Band edge alignment of monodoped anatase TiO_2 and $SrTiO_3$

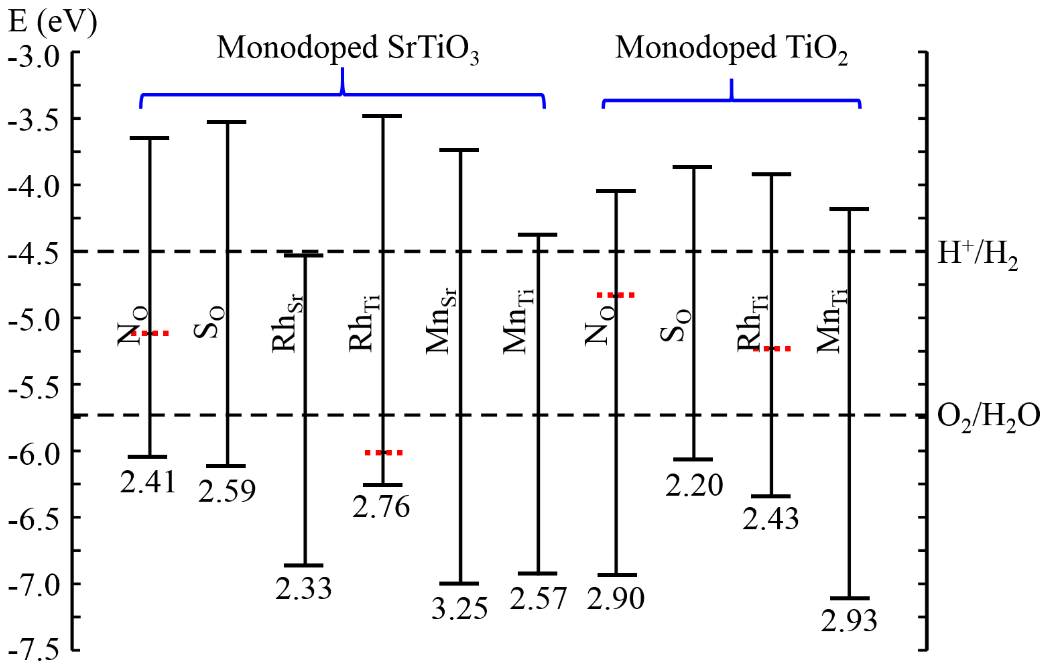

Figure S3: Band edge alignment of monodoped $SrTiO_3$ and TiO_2 w.r.t. water reduction and oxidation potential levels (H^+/H_2, O_2/H_2O). The dashed red line in forbidden region is representing the lowest unoccupied states.

V. Spin up and spin down bands of $Mn_{Ti}S_O$ codoped anatase TiO_2 and $Mn_{Sr}N_O$ codoped $SrTiO_3$

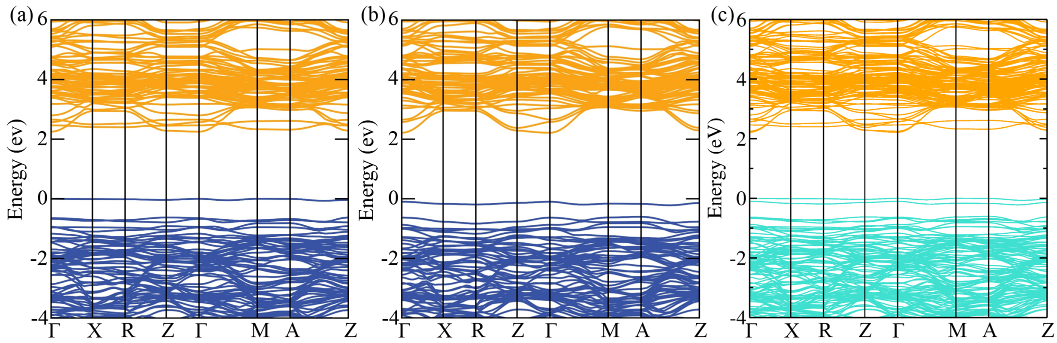

Figure S4: (a) Spin up, (b) spin down and (c) total band structures of $Mn_{Ti}S_O$ codoped TiO_2.

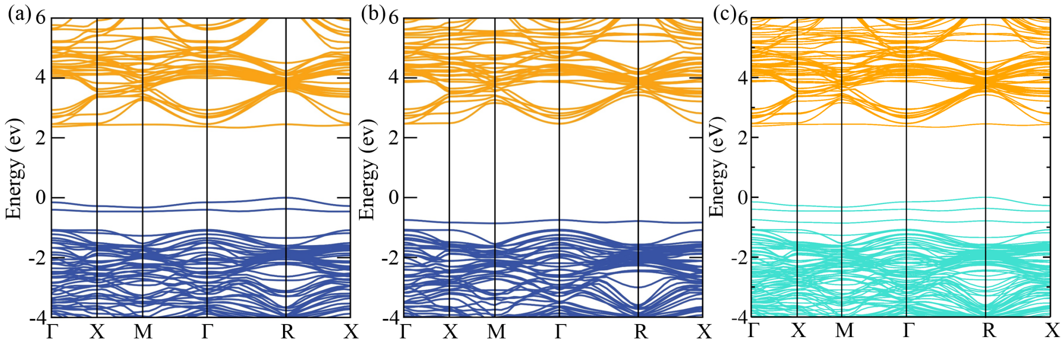

Figure S5: (a) Spin up, (b) spin down and (c) total band structures of $Mn_{Sr}N_O$ codoped $SrTiO_3$.

VI. Validation of supercell size of anatase TiO_2

Note that we have performed our entire calculations using HSE06 method. This is already quite expensive even for a 48 atoms supercell with periodic boundary conditions. But, we have not reduced our supercell to 48 atoms in order to save CPU hours. First we have checked from the band structure of a single Oxygen vacant supercell that the defect is fully localized (see the flat bands shown for the defect state in Figure S6(b)).

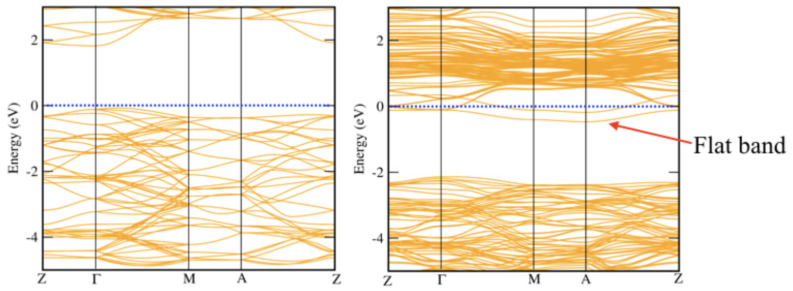

Figure S6: Band structure of (a) pristine anatase $Ti_{16}O_{32}$ and (b) with O-vacancy ($Ti_{16}O_{31}$).

In addition, we have done the single O-vacancy stability analysis at various charge states by varying the chemical potential of electron. We can see from Figure S7 below, the stability of the charge state does not get changed with the size of the supercell. For both 48-atom and 96-atom supercell, the following formation energy plot for single O-vacancy are identical (in terms of stability).

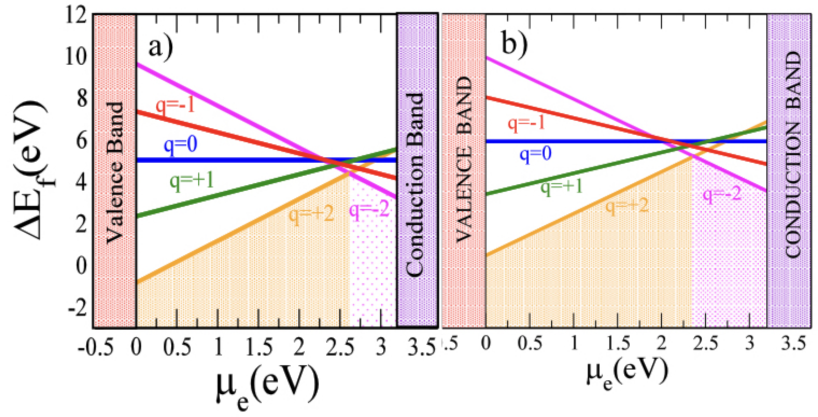

Figure S7: Formation energy plot for single O-vacancy with PBE functional for (a) 48-atom supercell and (b) 96-atom supercell.

This further establishes that even if we had increased the supercell size to 96 atoms, the stability of the charged defects would have not been affected at all.

In addition, we have also checked that the stability of charged defects in N-doped $SrTiO_3$ with different supercells (viz. 90-atom and 40-atom) remains same (using HSE06 functional).
